# *De novo* mutations in mental retardation

**DOI:** 10.1186/gb-2011-12-s1-i16

**Published:** 2011-09-19

**Authors:** Joris A Veltman

**Affiliations:** 1 Department of Human Genetics, Radboud University Nijmegen Medical Centre, PO Box 9101, 6500 HB Nijmegen, The Netherlands

## 

Recent studies have indicated that humans have an exceptionally high per-generation mutation rate of 7.6 × 10^–9^ to 2.2 × 10^–8^. These spontaneous germline mutations can have serious phenotypic consequences when affecting functionally relevant bases in the genome. In fact, their occurrence may explain why cognitive disorders with a severely reduced fecundity, such as mental retardation, remain frequent in the human population, especially when the mutational target is large and comprises many genes. This would explain a major paradox in the evolutionary genetic theory of these disorders. In this presentation, I will describe our recent work on using a family-based exome sequencing approach to test this *de novo* mutation hypothesis in ten patients with unexplained mental retardation [1]. Unique nonsynonymous *de novo* mutations were identified and validated in nine genes. Six of these, identified in different patients, were likely to be pathogenic based on gene function, evolutionary conservation and mutation impact. The clinical relevance of these novel genes, and the ultimate proof that they cause disease, lies in the identification of *de novo* mutations in additional patients with a similar phenotype. As such, we are currently screening approximately 1,200 patients with unexplained mental retardation for mutations in *YY1*, which is one of these newly identified genes. In addition, we are extending our family-based exome sequencing approach to 100 patients to establish the diagnostic yield for *de novo* mutations in patients with unexplained mental retardation. These findings, when replicated, provided strong experimental support for a *de novo* paradigm for mental retardation. Together with *de novo* copy number variation, *de novo* point mutations of large effect could explain the majority of all mental retardation cases in the population. In my presentation, I will explain this work, as well as related work on autism [2] and schizophrenia [3].

## References

[B1] Vissers LELM de Ligt J Gilissen C Janssen I Steehouwer M de Vries P van Lier B Arts P Wieskamp N del Rosario M van Bon BWM Hoischen A de Vries BBA Brunner HG Veltman JA A *de novo* paradigm for mental retardation Nat Genet 2010 42 1109 1112 10.1038/ng.712 21076407

[B2] O’Roak BJ Deriziotis P Lee C Vives L Schwartz JJ Girirajan S Karakoc E Mackenzie AP Ng SB Baker C Rieder MJ Nickerson DA Bernier R Fisher SE Shendure J Eichler EE Exome sequencing in sporadic autism spectrum disorders identifies severe *de novo* mutations Nat Genet 2011 43 585 589 10.1038/ng.835 21572417 PMC3115696

[B3] Girard SL Gauthier J Noreau A Xiong L Zhou S Jouan L Dionne-Laporte A Spiegelman D Henrion E Diallo O Thibodeau P Bachand I Bao JY Tong AH Lin CH Millet B Jaafari N Joober R Dion PA Lok S Krebs MO Rouleau GA Increased exonic de novo mutation rate in individuals with schizophrenia. Nat Genet 2011 43 860 863 doi: 10.1038/ng.886 21743468 10.1038/ng.886

